# Silica-Based Aerogel Composites Reinforced with Reticulated Polyurethane Foams: Thermal and Mechanical Properties

**DOI:** 10.3390/gels8070392

**Published:** 2022-06-21

**Authors:** Beatriz Merillas, Alyne Lamy-Mendes, Fernando Villafañe, Luisa Durães, Miguel Ángel Rodríguez-Pérez

**Affiliations:** 1 Cellular Materials Laboratory (CellMat), Condensed Matter Physics Department, Faculty of Science, University of Valladolid, Paseo de Belén 7, 47011 Valladolid, Spain; marrod@fmc.uva.es; 2 CIEPQPF, Department of Chemical Engineering, University of Coimbra, Rua Sílvio Lima, 3030-790 Coimbra, Portugal; alyne@eq.uc.pt (A.L.-M.); luisa@eq.uc.pt (L.D.); 3 GIR MIOMeT-IU Cinquima-Química Inorgánica, Faculty of Science, University of Valladolid, Paseo de Belén 7, 47011 Valladolid, Spain; fernando.villafane@uva.es; 4 BioEcoUVA Research Institute on Bioeconomy, University of Valladolid, 47011 Valladolid, Spain

**Keywords:** silica aerogel, polyurethane foam, composites, thermal insulation, reinforcement

## Abstract

In this work, silica aerogel composites reinforced with reticulated polyurethane (PU) foams have been manufactured having densities in the range from 117 to 266 kg/m^3^ and porosities between 85.7 and 92.3%. Two different drying processes were employed (ambient pressure drying and supercritical drying) and a surface modification step was applied to some of the silica formulations. These composites, together with the reference PU foam and the monolithic silica aerogels, were fully characterized in terms of their textural properties, mechanical properties and thermal conductivities. The surface modification with hexamethyldisilazane (HMDZ) proved to improve the cohesion between the reticulated foam and the silica aerogels, giving rise to a continuous network of aerogel reinforced by a polyurethane porous structure. The samples dried under supercritical conditions showed the best interaction between matrixes as well as mechanical and insulating properties. These samples present better mechanical properties than the monolithic aerogels having a higher elastic modulus (from 130 to 450 kPa), a really exceptional flexibility and resilience, and the capacity of being deformed without breaking. Moreover, these silica aerogel-polyurethane foam (Sil-PU) composites showed an excellent insulating capacity, reaching thermal conductivities as low as 14 mW/(m·K).

## 1. Introduction

Since the first synthesis of aerogels at the beginning of 1930s by Kistler [1,2], the interest for these excellent materials has been ever growing and further research is expected in the future [3]. Although there exist several types of aerogels, the most explored are the silica-based ones. Silica aerogels are highly mesoporous materials (>80% of porosity) with ultralow density, having huge internal surface areas (typically from 250 to 800 m^2^ g^−1^ and can exceed 1000 m^2^ g^−1^) [4], high visible transparency [5], and extremely low thermal conductivities (10–30 mW/(m·K)) [6].

One of the main drawbacks of silica aerogels is their poor mechanical properties, in particular their low toughness. Due to this, research on this topic is essential. This improvement of the mechanical performance can be based on the control of the internal network structure during the synthesis of the aerogel or on the addition of a reinforcing phase. The fragility of the internal structure is due to the thin necks joining the silica nanoparticles, and the brittleness of the O-Si-O bonds under an impact force [1,7,8]. The addition of carbon nanotubes (CNTs) has been used in several recent works such as the one of Lamy-Mendes et al. [9] who synthesized reinforced CNTs-silica aerogels with improved physical and mechanical properties. The obtained aerogel showed a higher Young’s modulus, reaching values above 201.5 kPa, higher porosities and lower densities than the pure aerogels. Another example is the work of Piñero et al. [10] in which functionalized CNTs are included into the silica formulations, achieving a significant stiffening effect on the resultant monoliths. Different reinforcing fibers have also been used in several works such as polymeric fibers [11,12,13], or glass fibers [14,15,16].

However, one of the main problems of using these strategies lies in the effect of the fibers/nanoparticles/CNT dispersion that could be heterogeneous, reaching in this way non-homogeneously reinforced silica monoliths. Therefore, solutions based on the formation of the silica aerogel into an already formed support were explored.

The fabrication of silica aerogels in combination with a carbon foam (CF) of 40–80 µm of pore size acting as solid skeleton (CF/SiO_2_ composite) was evaluated by Liu et al. [17] in 2019. These composites were fabricated through the sol–gel method and dried under atmospheric pressure. A significant improvement on the compressive stress of the CF/SiO_2_ composite was observed as well as a reduction in the thermal conductivity of the initial carbon foam (35 mW/(m·K)), yielding composites with improved thermal insulating properties (24 mW/(m·K) at room temperature). However, due to the poor interaction between the carbon and the silica matrix, not all the foam cells were filled with the aerogel and some cracks on the surface were present. Additionally, Ye et al. [18] fabricated a similar type of composite but coating the carbon foam by chemical vapor deposition with SiC to enhance the resistance of the skeleton matrix, and to prevent the aerogel from collapsing. Then, the silica aerogel was formed inside the support obtaining the SiC/CF–aerogel composite, whose compressive strength was greatly increased. Nevertheless, some aerogel fragments fell out of the carbon matrix because of the shrinkage during the ambient pressure drying. The thermal conductivity of the carbon foam was from 240 to 32 mW/(m·K) at 100 °C.

Herein, a strategy based on the silica aerogel fabrication into a reticulated polyurethane (PU) foam is presented. The advantage of the employed reinforcement matrix is twofold. Not only are PU foams well-known materials because of their versatility and interesting insulation and lightness properties, but they also provide a continuous solid network, avoiding heterogeneity and the dispersion step needed for other commonly used reinforcements for silica aerogels such as glass fibers [19] or pre-oxidized fibers [20]. The sol–gel method was employed for the aerogel synthesis and the obtained gels were dried under ambient pressure conditions and under supercritical drying. The carbonyl and amine groups of the polyurethane foam could stablish a chemical interaction with the silica matrix, promoting an effective interaction and, thus, improving the mechanical properties of the silica aerogel. Furthermore, an excellent insulating capacity is observed if the silica aerogel is able to fill the cells of the PU foams.

## 2. Results and Discussion

The final properties of the synthesized silica aerogel and Sil–PU composites have been deeply studied. The analysis of the different properties can be found in the following sections.

### 2.1. PU Foams and Silica Aerogels Characterization

**Figure 1:**
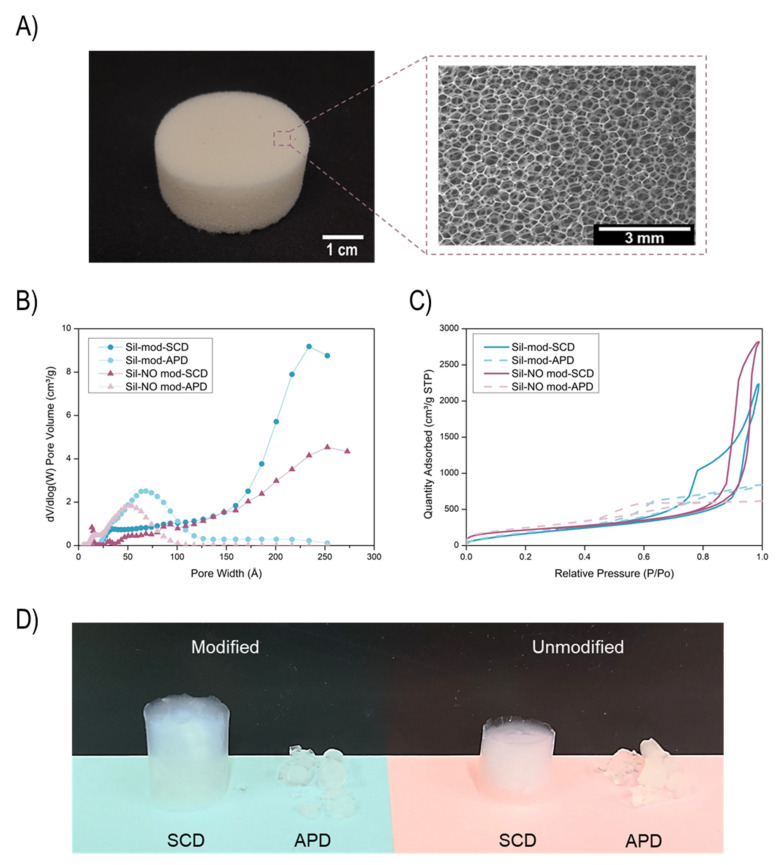
(**A**) Reticulated PU foam and its porous structure observed by a scanning electron microscopy. (**B**) Pore width distribution for the silica aerogels. (**C**) Nitrogen quantity adsorbed as a function of the relative pressure for the silica aerogels. (**D**) Picture of the synthesized silica aerogels.

A reticulated polyurethane foam was selected as a reinforcement of silica aerogels. The main structural feature of this type of foams is their solid interconnected skeleton formed by struts whose walls have been removed, as shown in Figure 1A. As gathered in Table 1, this foam shows a very low density, 29.4 kg/m^3^, and, thus, an extremely high porosity of 97.5%. The average cell size of the foam is 435 mm. Moreover, its thermal conductivity was measured, obtaining a value of 33 mW/(m·K). This is the expected value for a low-density open-cell polymeric foam in which the air in the cells with a conductivity of 26 mW/(m·K) at room temperature has a significant contribution to the total thermal conductivity.

A silica formulation based on tetraethylorthosilicate (TEOS) as the silica precursor was used for aerogel synthesis. The obtained aerogels were named Sil-APD or Sil-SCD depending on the drying method used for their obtention (APD means ambient pressure drying and SCD supercritical drying). In some of the gels, a modification was performed before the drying step by using hexamethyldisilazane (HMDZ). This reagent makes alcogels hydrophobic and, therefore, minimizes the hygroscopicity of the final aerogels and also the shrinkage during APD. The aerogels which were modified were labeled as Sil-APD m and Sil-SCD-M. The main properties of the aerogels can be found in Table 1. An increase in the final density of aerogels dried under ambient pressure conditions (xerogels) was observed, which leads to a noticeable decrease in the porosity and to a significant increment of the thermal conductivity. However, APD aerogels present a larger specific surface area and the consequent reduction in the average pore size. This fact is mainly due to the higher shrinkage that the solvent evaporation promotes during the ambient pressure drying. Still, APD aerogels always show porosity above 82%.

Graphs B and C of Figure 1 show the pore size distribution and the nitrogen amount adsorbed by the aerogel samples, respectively. The effect of the pore size reduction for the APD aerogels having pore sizes around 5–7 nm can be clearly seen, whereas the SCD ones present average peaks at higher values (22–25 nm), slightly reducing the specific surface area. Additionally, supercritically dried aerogels adsorb a higher amount of nitrogen owing to the higher porosity caused by the lower shrinkage of these samples.

The implemented modification contributes to the improvement of some of the aerogel properties. When the HMDZ modification is performed, silica aerogels have a lower density, reaching values as low as 79.2 kg/m^3^ because the shrinkage is further decreased. This effect was previously analyzed by Torres et al. [14] when different silylating agents were employed for surface modification. Moreover, the modified aerogels present the lowest thermal conductivity, being reduced from 52.2 to 32.7 mW/(m·K) for APD and 20.9 to 17.3 mW/(m·K) for SCD.

In Figure 1, picture D, the synthesized monolithic silica aerogels (without foam) are depicted. When SCD is performed, consistent monoliths can be obtained. Nevertheless, aerogels dried under ambient pressure conditions were so brittle that they were broken into small pieces during their handling.

**Figure 2:**
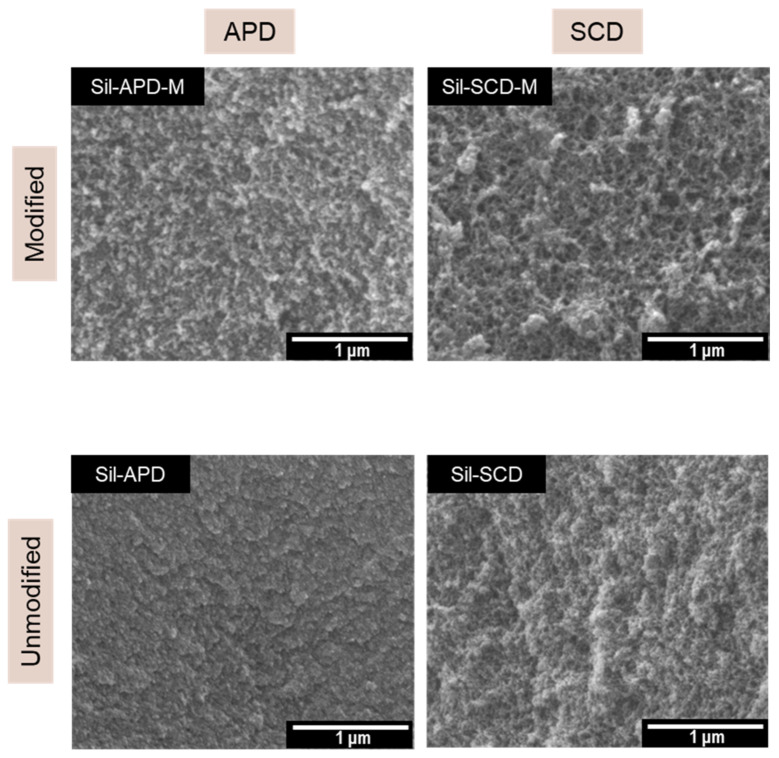
Scanning electron micrographs for the different silica aerogels: Sil-APD, Sil-SCD, Sil-APD m and Sil-SCD-M.

The scanning electron micrographs obtained for the silica aerogels are displayed in Figure 2. Regarding the influence of the drying process, it is noticeable that porosity decreases when aerogels were obtained from APD due to the strong shrinkage experimented during drying. Moreover, these samples have smaller-sized particles and pores, as explained before, thus showing a more compact structure. However, SCD composites exhibit larger pores and porosities since the drying procedure did not significantly affect their porous structures.

Additionally, the influence of the surface modification can be noticed since the unmodified aerogels present smaller particles (larger surface areas were obtained) and highly agglomerated silica particles.

### 2.2. Composite Characterization

**Figure 3:**
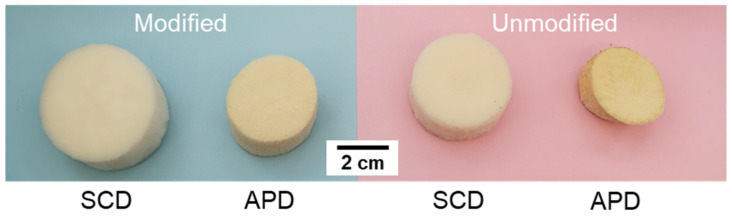
Sil–PU composites obtained by performing an HMDZ modification (blue color) or without modification (pink color).

Once the silica sols were synthesized with the developed silica formulations, they were poured into a mold which contains a PU foam. The solution was poured into the inner pores until the polyurethane foam was completely filled. Moreover, the polymer matrix forming the foam swelled the absorbing part of the gel solution until gelation occurred. In this way, the size of the gel composite after the gelation time was higher than that of the initial foam. After the washing, modification (when applied) and drying steps, the final composites were obtained (Figure 3). It was visually noticed that APD composites underwent stronger shrinkage than when supercritical drying was employed, the same result that was observed for the silica monoliths. The obtained Sil–PU composites are characterized in the following sections.

#### 2.2.1. Density, Shrinkage and Porosity

The bulk densities of the synthesized composites are gathered in Table 2. As expected, the same effect, previously analyzed for the silica aerogels, was observed: SCD composites showed lower densities than the APD ones and the silylated composite reached the lowest density of 117.68 kg/m^3^. There was a remarkably increase in the density of the obtained composites in comparison with the polyurethane foam density, which is due to the formation of a 3D network of silica aerogel filling the inner pores. Additionally, the porosity of the Sil–PU composites was calculated, finding larger porosities for the composites dried under supercritical conditions (92.3 and 91.5%), whereas the APD composites presented smaller porosities (89.9 and 85.7%).

Linear and volumetric shrinkages were measured taking into account the initial size of the PU foam and the final size of the dried composite. Since the PU foam absorbs a significant amount of solvent during the gelation step, when the shrinkages are not significantly large, these values can be negative, as is the case of the composite C-SCD-M. This sample, due to the supercritical drying and the applied surface modification which improves the cohesion between both matrixes, undergoes the smallest amount of shrinkage (smaller than the swelling expansion) and, therefore, its final linear shrinkage is negative. Once again, the influence of the drying method is clear: the APD method leads to a high shrinkage, contributing to the increase in the final density. Furthermore, shrinkages were minimized for the modified samples since the HMDZ prevents the silanol groups from condensing [21].

The highest shrinkage was found for the composite C-APD (32 and 59% for linear and volumetric shrinkages, respectively). However, when the same composite was dried under supercritical conditions, these shrinkage values were sharply reduced to 7 and 12%, respectively. When the modification was performed, these values further diminished, being around 20% for the APD sample and even negative for the composite C-SCD-M.

The percentage of aerogel mass which is incorporated into each composite was calculated as the weight difference between the initial foam and the final composite. There is a significant effect of modifying the aerogel which remains inside the PU foam owing to additional interactions between the silica aerogel and the reticulated foam. The values for the modified samples (C-SCD m and C-APD-M) are 82 and 87%, while, with no modification, these values decreased to76 and 74%, indicating a lower filling of the PU foam’s internal structure. It has to be explained that the higher amount of aerogel that is present in the modified composites does not lead to a higher density, hence the reduced shrinkage of these samples, which causes their lightweight behavior.

#### 2.2.2. Porous Structure

**Figure 4:**
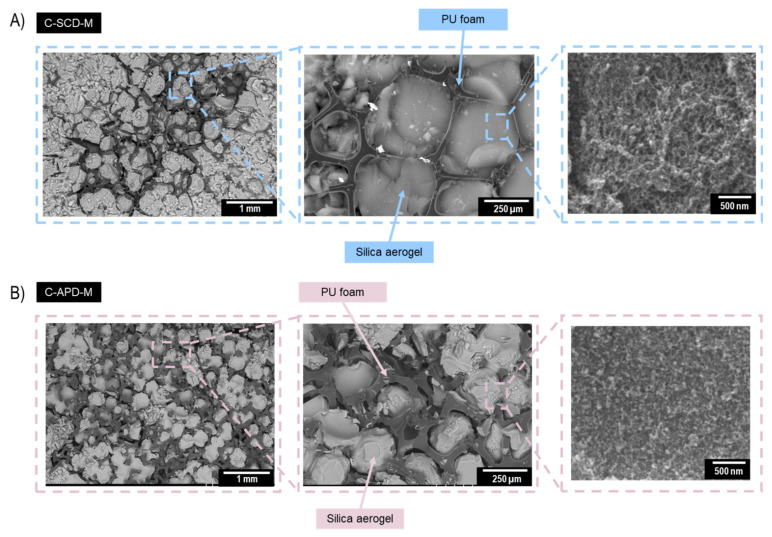
Scanning electron micrographs of the composite: (**A**) C-SCD-M and (**B**) C-APD-M.

The inner structure of the fabricated composites was analyzed by means of a backscattered electron detector, as shown in Figure 4. The micrographs on the left side show a general image of the composites C-SCDM (up) and C-APD-M (down). For the SCD and modified sample, several individual pores filled by the silica aerogel can be distinguished.

The effective cohesion between the polyurethane struts and the silica aerogel matrix can be observed in Figure 4A. The silica matrix can chemically interact with the carbonyl and amine groups present in the polyurethane foam. Therefore, there exist minor aerogel breaks in some of the pores, although most of them are completely filled, forming a continuous silica aerogel network.

Nevertheless, the APD composite (Figure 4B) revealed several aerogel breakages caused by the shrinkage during the drying step. These breakages contribute to the increase in the disconnections between the polyurethane struts and the silica aerogel. The SEM micrographs for the other composites can be found in the Supporting Information, Figure S1.

#### 2.2.3. Mechanical Properties

**Figure 5:**
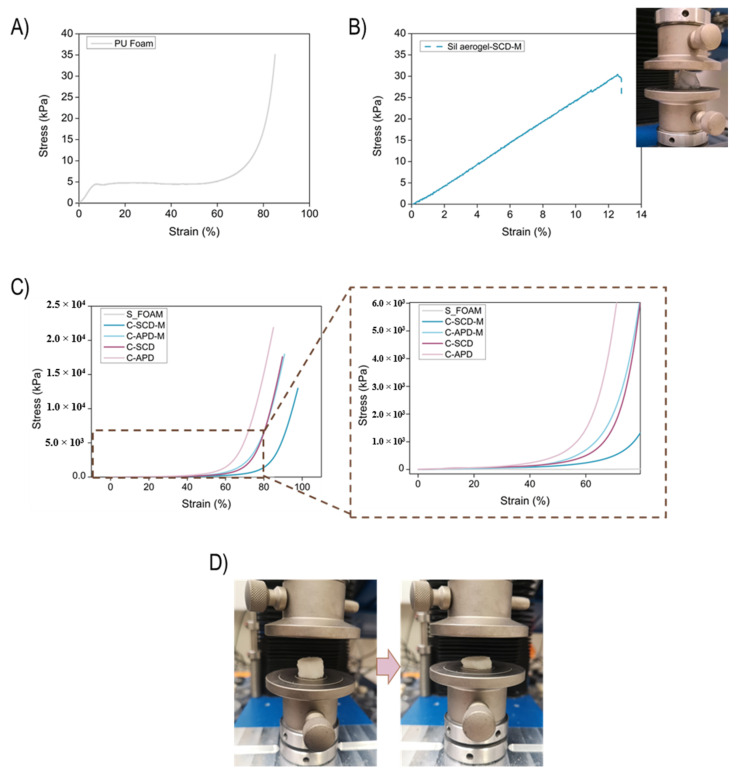
(**A**) Stress–strain curve for the polyurethane foam. (**B**) Stress–strain curve for one of the monoliths (Sil-SCD-M) until break. (**C**) Stress–strain curve for all the composites obtained from the destructive tests. (**D**) Picture of sample C-SCD-M before and after the compression test.

Compression–decompression cycles were carried out in order to evaluate the elasticity of the manufactured composites and their elastic moduli. Additionally, samples were tested until reaching high deformations (destructive tests). Figure 5A shows a destructive experiment for the PU foam. There exists a linear region until 10% strain; then, a plastic deformation plateau can be observed from 10 to 60% of strain, and the final densification step of the foam occurs from 60% deformation on, reaching a maximum strain of ca. 80% in the limit of the test.

The SCD-modified monolithic silica aerogel experiment is plotted in Figure 5B. After the linear deformation, when the sample reached a strain of c.a. 12%, the aerogel was broken into small pieces (30 kPa).

The stress–strain curves for the Sil–PU composites are displayed in Figure 5C. These composites allow higher deformations than the reference silica monoliths to be reached. Furthermore, there exists a clear trend: when composites are dried under supercritical conditions, they can be deformed more than the corresponding APD composites. This is mainly due to the lower bulk density and the continuous solid aerogel network which is included into the PU foam when SCD is applied. Additionally, it is noticeable that none of the composites under study was broken, accounting for the enhanced compression endurance. On the contrary, they were strongly densified.

The previous break observed for the monolithic aerogel was not observed when the aerogel was reinforced with the polyurethane matrix, reaching flexible composites that are only permanently and irreversibly deformed when a strain higher than 40% is applied (Figure 5D).

**Figure 6:**
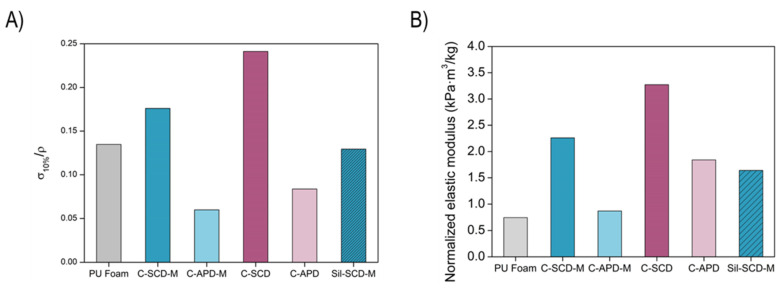
(**A**) Stress at a 10% of strain normalized with the bulk density. (**B**) Normalized elastic modulus.

The stiffness that the samples present under compression was assessed and compared by the calculation of the stress at a strain of 10%. In order to remove the effect that density has on this parameter, these values were normalized by the corresponding density, as displayed in Figure 6A. The APD samples showed a significant stiffness, taking into account that the corresponding silica monoliths (Sil-APD-M and Sil-APD) did not remain in monolithic form after being dried. The same occurred for the non-modified SCD composite (C-SCD), whose corresponding silica monolith was broken. In the case of the SCD-modified formulation, a significant improvement in the stiffness was reached for the composite (C-SCD-M) in comparison with the silica monolith (Sil-SCD-M).

Moreover, the elastic modulus was calculated from the linear region of the curves (2–4% for foams and 5–9% for the composites) (numerical values can be found in Supporting Information, Table S1). The obtained values were also corrected with the corresponding density in order to establish a comparison between samples and are plotted in Figure 6B. These results are in agreement with the stress at 10% of deformation, with the composites being stiffer than the silica monoliths. Regarding the numerical value of the normalized elastic modulus, the SCD composites showed the highest compression modulus, reaching relative values of 2.26 (C-SCD-M) and 3.27 kPa·m^3^/kg (C-SCD). When comparing the composite- and silica monolith-normalized elastic moduli, a remarkable improvement can be observed, an increase from 1.64 kPa·m^3^/kg for the silica aerogel to 2.26 kPa·m^3^/kg for the corresponding composite. The APD still present a normalized elastic modulus higher than the initial foam (0.74 kPa·m^3^/kg) and the silica monolith with the same formulations (broken), proving the relevance of reinforcing the aerogels through a reticulated matrix.

**Figure 7:**
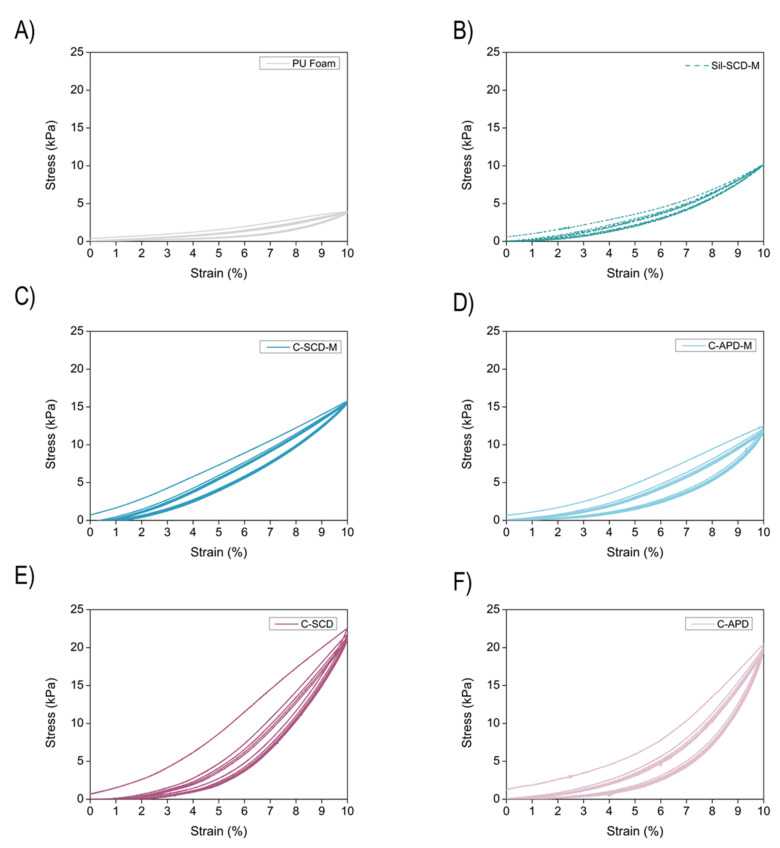
Uniaxial cyclic compression–decompression tests (5 cycles) at a strain of 10% for (**A**) the PU foam, (**B**) the modified SCD silica aerogel, and the composites (**C**) C-SCD-M, (**D**) C-APD-M, (**E**) C-SCD, (**F**) C-APD.

Then, a total of five cycles at a strain of 10% were performed, as shown in Figure 7. The polyurethane foam (grey color) presents a clear elastic behavior, reaching 10% strain with a stress of less than 5 kPa. The silica aerogel (dotted line) that was tested (Sil-SCD-M) also showed an elastic but stiffer behavior (10 kPa for 10% strain). In the case of the Sil–PU composites, elasticity is still the main feature since almost the same height was reached after the five compression–decompression cycles. Moreover, these composites require a higher stress for deformation by 10% of their initial height due to their higher density. The composites that were not modified (C-SCD and C-APD, pink color) showed the highest stiffness with a maximum stress of 20 kPa for the selected strain. Nevertheless, the maximum stress for deforming the modified composites (C-SCD-M and C-APD-M, blue color) was lower, being around 15 KPa.

**Figure 8:**
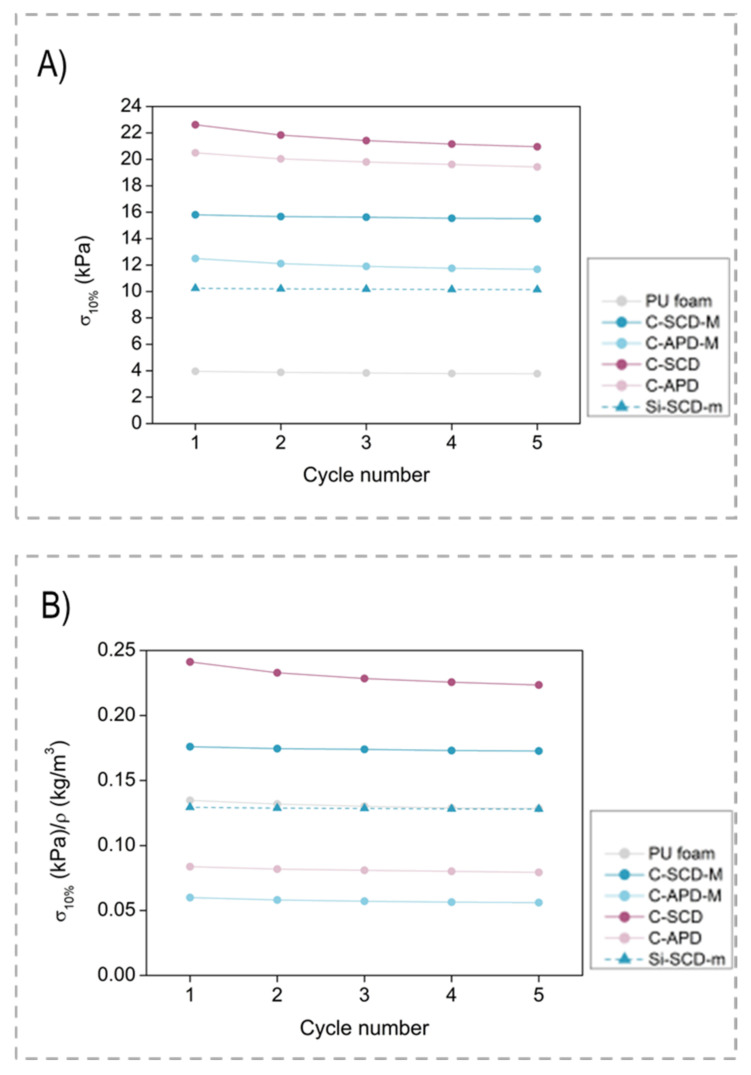
Stress at 10% strain for all the samples measured at each compression–decompression cycle: (**A**) absolute values; (**B**) relative values normalized with density.

From each cycle, the stress at a 10% deformation can be calculated to assess if any variation occurred during the experiment. Figure 8 depicts the absolute values of stress (Figure 8A) and relative values when corrected with the corresponding density (Figure 8B) for the four composite samples, the pure foam and one of the silica aerogels (Sil-SCD-M) (numerical values can be found in Supporting Information, Tables S2 and S3). The absolute and relative values for stress are rather similar for the five compression-decompression cycles, staying constant during all the experiment cycles. Since density increased during the composite formation, all of the composites showed the highest absolute values of stress (Figure 8A). However, once these values were normalized by applying the density correction (Figure 8B), the comparison between the composite C-SCD-M and the silica monolith Sil-SCD-M with the same formulation was still favorable. Therefore, the stiffness enhancement reached for the Sil–PU composites remains when compression–decompression cycles are applied, accounting for the samples’ flexibility.

Taking into account the previous data, these composites can be deformed several times until a 10% of strain without significantly altering their porous structure and mechanical performance, highlighting an exceptional flexibility and deformation capacities much higher than the reference monolith, which breaks at a strain of 12%.

#### 2.2.4. Thermal Conductivity

**Figure 9:**
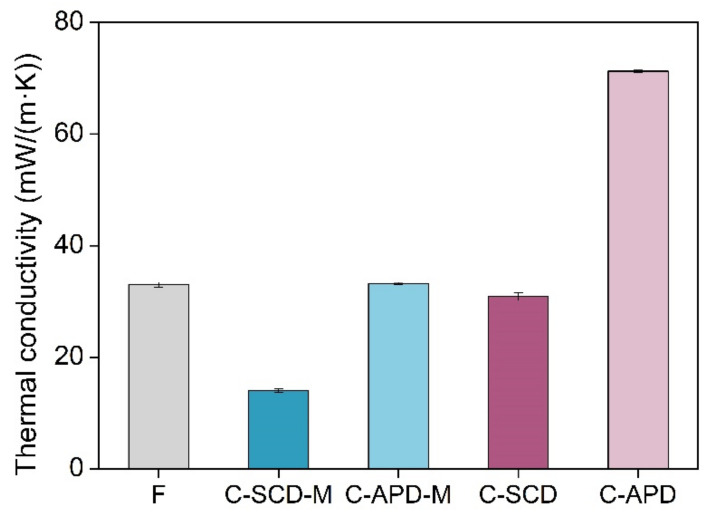
Thermal conductivity for the composite samples.

Finally, the insulating capacity of the manufactured samples was analyzed. Figure 9 shows the thermal conductivity of the composite samples. At 10 °C, the thermal conductivity of the initial PU foam was 33 mW/(m·K).

The contributions to the effective thermal conductivity have to be taken into account to explain the obtained results. The total thermal conductivity (*λ*_T_) is the result of the addition of several contributions:

$$\lambda_{T}=\lambda_{s}+\lambda_{g}+\lambda_{r}$$

where *λ*_s_ and *λ*_g_ are the conduction through the solid and gaseous phases, respectively, and *λ*_r_ is the radiation contribution.

The solid conduction took place through the struts of the reticulated foam in the case of the PU reference foam. For the Sil–PU composites, an additional solid phase was included, thus increasing density and the corresponding solid conduction. However, this increase is minimal since the solid contribution of the silica nanoparticles was really low, a few mW/(m·K).

The conduction through the gaseous phase is the main contribution to the total thermal conductivity for the PU foam since its pores are in the micrometric scale and the gas molecules are able to effectively collide and transfer the heat between them. However, the silica aerogels present tiny pores, far smaller than the mean free path of the air molecules at ambient pressure (70 nm). Therefore, the well-known Knudsen effect [22] takes place in these materials and the gaseous conduction will be strongly reduced. The reinforced composites have pores similar to those of the reference aerogels, although possible breaks can be present. For these reasons, the obtained thermal conductivities are slightly larger than those of the pure aerogels; the APD composites show the highest conductivities, 71.3 mW/(m·K) for the non-modified sample (C-APD) (versus 52.2 mW/(m·K) for the corresponding aerogel) and 33.2 mW/(m·K) for the modified composite (C-APD-M). The latter shows almost the same insulating properties than the pure silica aerogel (Sil-APD m 32.7 mW/(m·K)). This fact is mainly due to the effect that the HMDZ modification has aerogel integrity when formed into the PU foam. The SCD composites confirm this statement: the C-SCD composite shows a thermal conductivity of 30.9 mW/(m·K) (versus the 20.9 mW/(m·K) of Sil-SCD) and, when the modification is performed, this value is significantly reduced, reaching a value as low as 14.0 mW/(m·K). It has to be noted that the silica aerogels were measured in powder form, so the monolithic thermal conductivity will be slightly lower.

The radiative heat transfer depends directly on the relative density of the samples. Hence, the APD composites present a higher radiation contribution in comparison with the weight of the solid and gaseous contributions, but it is not a relevant term giving the temperature of the measurements.

To conclude, the supercritically dried modified composite exhibited the best insulating performances, reaching a thermal conductivity of 14 mW/(m·K).

## 3. Conclusions

**Figure 10:**
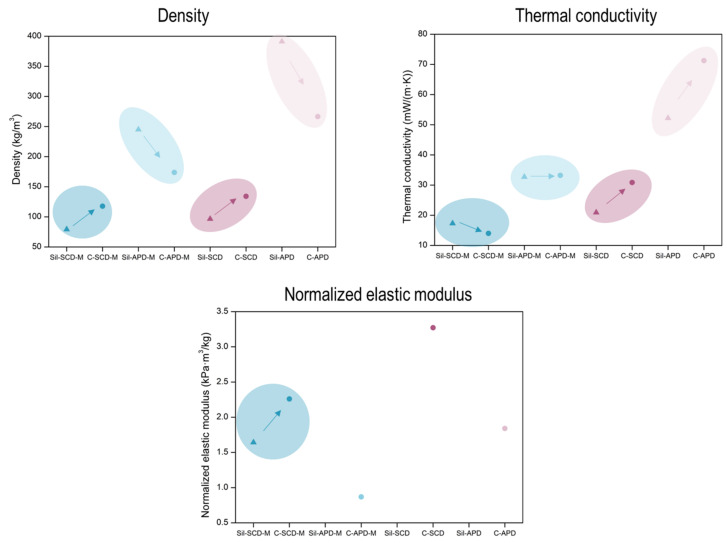
Main properties of the synthesized composites (C) and pure aerogels (Sil).

Silica composites reinforced with a reticulated polyurethane foam were synthesized by using the sol–gel method. Different types of drying (APD and SCD) were employed and a surface modification with HMDZ was implemented to study its effect on the final properties (a summary is gathered in Figure 10).

Firstly, the PU foam and the pure silica aerogels were fully characterized and, once the composites were obtained, their density, shrinkage, aerogel mass, porous structure, mechanical and thermal properties were assessed.

The APD composites presented the highest densities owing to a larger shrinkage during the drying step, while the SCD composites showed the lowest densities and, thus, the greatest porosities. Moreover, the silylation modification further reduced the final density, reaching a value of 117 kg/m^3^. This modification also promotes a more stable aerogel structure by improving the connection between both matrixes, as seen in the SEM images. This fact was also observed in the aerogel mass percentage that is included into the final composites (reaching 85% for the modified samples).

The effects of the drying procedure and the modification were also analyzed in the mechanical properties and thermal conductivities of the final composites.

The compression–decompression tests indicated that the composites were significantly stiffer than the monolithic aerogels while maintaining excellent flexibility and resilience after five cycles at a strain of 10%. The elastic modulus was calculated and normalized with the bulk density, accounting for the increment in the aerogel stiffness when the PU reinforcement was performed. Moreover, the SCD composites could be deformed to a larger extent than the APD composites because of their better integrity. All the composites reached deformations of more than the 80% without breaking, whereas the pure aerogel was broken at 12% of strain.

The thermal conductivities reached by the composites revealed that the SCD and the performed modification improved the final properties of the aerogels. In this way, the SCD composites showed the lowest thermal conductivities, achieving a great insulating performance for the modified composite (C-SCD-M), 14 mW/(m·K).

## 4. Materials and Methods

### 4.1. Materials

Tetraethylorthosilicate (TEOS, Si(OC_2_H_5_)_4_, 98%) was purchased from Acros Organics, Geel, Belgium; ethanol (EtOH, absolute, C_2_H_5_OH), oxalic acid (C_2_H_2_O_4_, 99%) and ammonium hydroxide (NH_4_OH, 25% NH_3_ in H_2_O) were supplied by Fluka Analytical (Fluka Chemie GmbH, Buchs, Switzerland); and hexamethyldisilazane (HMDZ, (CH_3_)_3_SiNHSi(CH_3_)_3_, >98%) was obtained from Alfa Aesar (Thermo Fisher GmbH, Kandel, Germany). High-purity water was used to prepare the solutions of oxalic acid (0.01 M) and ammonium hydroxide (1 M) catalysts.

A reticulated polyurethane foam was provided by Recticel Ibérica, S.L. (Barcelona, Spain). This foam is an open-cell material in which the cell walls have been completely removed of the cellular structure. So, the solid phase of the foam is only formed by struts. Due to this particular structure, the filling with the silica aerogel was facilitated. The density of the foam was 29.4 kg/m^3^, the porosity was 97.5% and the average cell size was 435 µm.

### 4.2. Synthesis of Silica Aerogels and Silica–PU Composites

Firstly, a solution of the silica precursor (TEOS) was prepared by using ethanol as solvent. Then, the hydrolysis step was promoted by an acid catalyst based on oxalic acid (0.01 M) and the mixture was stirred at 300 rpm for 30 min with a magnetic stirrer. After 24 h at 27 °C, condensation was initialized by the ammonium hydroxide catalyst by stirring for 45 s at 300 rpm. In the case of the Sil–PU composites, the obtained sol was poured into a beaker, which contained the corresponding PU foam, until it was covered completely. For both reference silica aerogels and composites, after the gelation time, samples were placed into a lab oven at 27 °C for 7 days for aging. Finally, two washings with ethanol (2 × 12 h at 50 °C) were performed. In the case of the modified samples, surface modification of the gels was carried out with a mixture of HMDZ in EtOH (30% vol.) at 50 °C for 24 h.

The synthesized alcogels were dried using two different routes. Atmospheric drying for 5 h at room temperature, followed by 24 h at 60 °C and a final heating of 2 h at 150 °C, was carried out to obtain the APD samples. Supercritical drying (SCD) using CO_2_ was performed for the SCD samples at a temperature of 40 °C, with a pressure of 110 bar. Prior to the SCD drying step, samples were covered with ethanol to prevent premature evaporation. Samples with different dimensions were synthesized: cylindrical samples with a diameter between 12 and 16 mm and a height of ca. 10 mm for the mechanical tests and a diameter between 30 and 45 mm and a thickness between 20 and 22 mm for the thermal conductivity measurements.

### 4.3. Characterization Techniques

The effect of filling the PU foam pores with silica aerogel, as well as the employed drying process (APD or SCD), and chemical modification with HMDZ was assessed by measuring the characteristics of the composites: bulk density, porosity, volumetric and linear shrinkages, microstructure, thermal conductivity and mechanical properties.

#### 4.3.1. Density, Porosity and Aerogel Mass

For the bulk density (*ρ*_b_) calculation, the weight (AT261 MettlerToledo balance) and volume of the samples were measured.

Porosity (*Π*) was calculated for the reference aerogels using Equation (1):

(1) $$\Pi_{aerogel}=\left(1-\rho_{r}{}_{aerogel}\right)\cdot 100$$

where *ρ*_r aerogel_ is the relative density calculated as the ratio between the aerogel density (*ρ*_b_) and the solid density (ca. 2200 kg/m^3^) [23,24].

Porosity was also calculated for the Sil–PU composites through Equation (2):

(2) $$\Pi_{composites}=\chi_{aerogel}\cdot \;\Pi_{aerogel}\cdot 100$$

where *χ*_aerogel_ is the volume fraction of aerogel included in the composite, which corresponds to the porosity of the PU foam.

For the Sil–PU composites, the aerogel mass percentage was calculated as described by the following equation:

(3) $$\mathrm{aerogel}\;\mathrm{mass}\;\left(\%\right)=\frac{\left(m_{\mathrm{composite}}-m_{\mathrm{foam}}\right)}{m_{\mathrm{composite}}}\cdot 100$$

where *m*_composite_ and *m*_foam_ are the corresponding mass of the composite and initial foam.

#### 4.3.2. Volumetric and Linear Shrinkage

The volumetric and linear shrinkages were determined by comparing the volume of the foam (*V*_0_) and diameter (*d*_0_) prior to gel formation and the same characteristics of the composites (*V* and *d*, respectively) after the drying process by using Equations (4) and (5).

(4) $$S_{v}\left(\%\right)=\left(1-\frac{V}{V_{0}}\right)\cdot 100$$

(5) $$S_{l}\left(\%\right)=\left(1-\frac{d}{d_{0}}\right)\cdot 100$$

#### 4.3.3. Scanning Electron Microscopy

The monolithic aerogel samples were cut and metalized through an iridium sputter coater (EMITECH K575X Sputter Coater). Scanning electron micrographs were obtained by using an ESEM Scanning Electron Microscope (QUANTA 200 FEG, Hillsboro, OR, USA).

Composite samples were cut and metalized through a golden sputter coater and micrographs were obtained using a scanning electron microscope (FlexSEM 1000, Hitachi, Tokyo, Japan) and using a BackScattered Electron Detector (BSE).

#### 4.3.4. Specific Surface Area and Pore Size

The specific surface areas were measured by nitrogen sorption with a Micromeritics (Norcross, GA, USA) model ASAP 2020 instrument at the University of Malaga (Andalusia, Spain). Samples were degassed under high vacuum at 50 °C for 24 h. The Brunauer–Emmett–Teller (BET) [25] method was employed for the calculations. The pore size was determined by the Barrett–Joyner–Halenda (BJH) method through the adsorption branch of the isotherms.

#### 4.3.5. Mechanical Properties

Uniaxial compression tests were carried out for the composite samples (cylindrical samples with a diameter between 12–and 16 mm and a height of around 10 mm) by using an Inspekt mini-series (Hegewald & Peschke, Nossen, Germany), with a strain rate of 1 mm/min. First, five compression–decompression cycles were performed with a strain of 10% and a loadcell of 50 N. The pre-load used for all the composites was ca. 0.1 N. Then, an additional compression–decompression cycle was carried out with a composite replicate under the same conditions but reaching a strain of 25% to evaluate the elastic modulus. Finally, destructive tests were also performed for the composites by using a loadcell of 3 kN until reaching a maximum load of 2.8 kN.

#### 4.3.6. Thermal Conductivity

Thermal conductivity was measured using a thermal heat flow meter model FOX 314 (TA Instruments/LaserComp, Inc, New Castle, DE, USA), which measures according to ASTM C518 [26] and ISO 8301 [27]. Due to the composite’s surface area, which is smaller than the area of the FOX 314 heat flux transducer (100 × 100 mm^2^), an external heat flux sensor gSKIN^®^ XM 27 9C (greenTEG AG) was employed to obtain the experimental heat flow in combination with a data logger gSKIN^®^ DLOG-4219 (greenTEG AG, Rümlang, Switzerland). The equipment cavity was filled with masks (EVA foams). Temperature was monitored by two thermocouples during the experiment. The thermal conductivity measurements were carried out at 10 °C with cylindrical samples having a diameter between 30 and 45 mm and a thickness between 20 and 22 mm.

Due to the difficulty of handling silica aerogels, their thermal conductivity was measured in powder form. Aerogels were first grinded and placed into the measurement EVA masks acting as a mold with dimensions of ca. 4 cm of diameter.

## Figures and Tables

**Table 1:** Main properties of the polyurethane foam and synthesized silica aerogel composites.

Properties	PU Foam	Sil-SCD-M	Sil-APD-M	Sil-SCD	Sil-APD
Density (kg/m^3^)	29.4 ± 0.7	79.2 ± 2.7	245.1 ± 36.8	96.5 ± 7.8	391.2 ± 31.5
Porosity (%)	97.5	96.4	88.9	95.6	82.2
BET Surface Area (m^2^/g)	-	700.0	760.2	781.3	912.8
BJH pore width (nm)	-	22	7	25	5
λ (mW/(m·K))	33.0 ± 0.4	17.3 ± 1.4	32.7 ± 1.0	20.9 ± 2.4	52.2 ± 3.0

**Table 2:** Composites properties.

Properties	PU Foam	C-SCD-M	C-APD-M	C-SCD	C-APD
Density (kg/m^3^)	29.39 ± 0.74	117.68	173.79	134.08	266.48
Porosity (%)	97.4	92.3	89.8	91.5	85.7
Linear Shrinkage (%)	-	−8.38	19.97	6.95	32.03
Volumetric Shrinkage (%)	-	−30.82	36.06	12.46	58.78
Aerogel mass (%)	-	82.05	86.95	76.02	74.20
λ (mW/(m·K))	33.00 ± 0.44	14.00 ± 0.33	33.22 ± 0.19	30.87 ± 0.69	71.26 ± 0.29

## Supplementary Materials

The following supporting information can be downloaded at: https://www.mdpi.com/article/10.3390/gels8070392/s1, Figure S1: Scanning electron micrographs for the unmodified composites (C-SCD and C-APD); Table S1: Elastic modulus for the produced samples; Table S2. Absolute values of stress at 10% of strain for all the samples measured at each com-pression-decompression cycle; Table S3. Relative values of stress at 10% of strain for all the samples measured at each compres-sion-decompression cycle.

## Abbreviations

APD	Ambient pressure drying
C-APD	Sil–PU composite dried at ambient pressure non-modified
C-APD-M	Sil–PU composite dried at ambient pressure and modified
CF	Carbon foam
CNTs	Carbon nanotubes
C-SCD	Sil–PU composite dried supercritically non-modified
C-SCD-M	Sil–PU composite dried supercritically and modified
HMDZ	Hexamethyldisilazane
PU	Polyurethane
SCD	Supercritical drying
Sil-PU	Silica aerogel-polyurethane foam composite
TEOS	Tetraethylorthosilicate
