# Supplementary material for: Silica-Based Aerogel Composites Reinforced with Reticulated Polyurethane Foams: Thermal and Mechanical Properties
Source: Gels. 2022 Jun 21;8(7):392. doi: 10.3390/gels8070392 (PMC9318614; doi:10.3390/gels8070392)
Supplement: Supplementary file 1 [file gels-08-00392-s001.zip › gels-1756004-supplementary.pdf]

gels

Article

Silica-Based Aerogel Composites Reinforced with Reticulated Polyurethane Foams: Thermal and Mechanical Properties

Beatriz Merillas [1,*], Alyne Lamy-Mendes [2], Fernando Villafañe [3], Luisa Durães [2] and Miguel Ángel Rodríguez-Pérez [1,4]

1 Cellular Materials Laboratory (CellMat), Condensed Matter Physics Department, Faculty of Science, University of Valladolid, Paseo de Belén 7, 47011 Valladolid, Spain; marrod@fmc.uva.es
2 University of Coimbra, CIEPQPF, Department of Chemical Engineering, University of Coimbra, CIEPQPF, Rua Sílvio Lima, 3030-790 Coimbra, Portugal; alyne@eq.uc.pt (A.L.-M.); luisa@eq.uc.pt (L.D.)
3 GIR MIOMeT-IU Cinquima-Química Inorgánica, Faculty of Science, University of Valladolid, Paseo de Belén 7, 47011 Valladolid, Spain; fernando.villafane@uva.es
4 BioEcoUVA Research Institute on Bioeconomy, University of Valladolid, 47011 Valladolid, Spain
* Correspondence: b.merillas@fmc.uva.es

Supporting Information

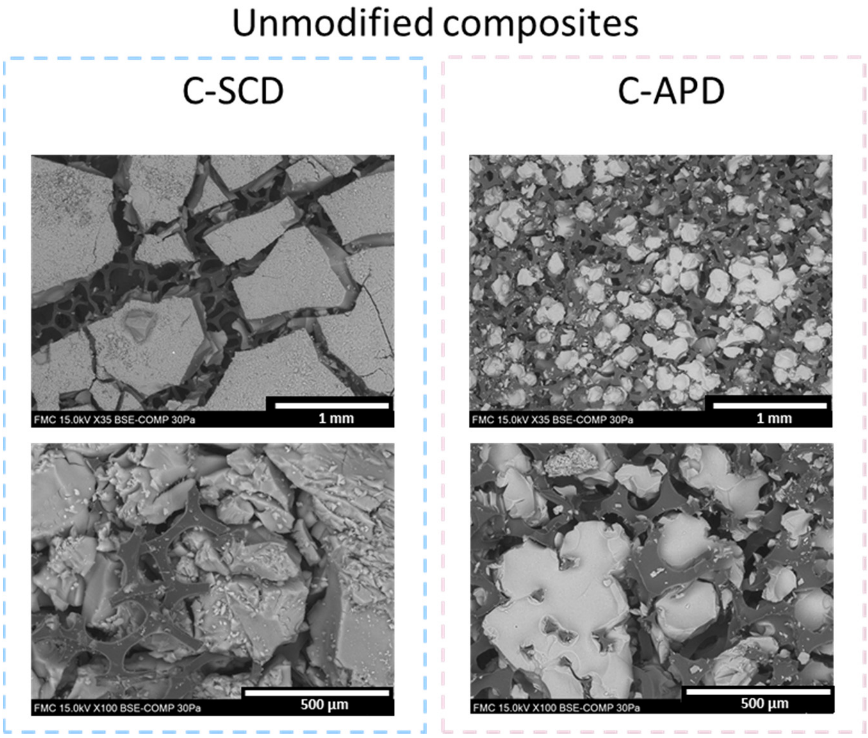

Figure S1. Scanning electron micrographs for the unmodified composites (C-SCD and C-APD).

Table S1. Elastic modulus for the produced samples.

Sample	Elastic modulus (kPa)
PU foam	21.87
C-SCD-M	202.98
C-APD-M	181.34
C-SCD	306.8
C-APD	451.03
Sil-SCD-M	130.00

Table S2. Absolute values of stress at 10 % of strain for all the samples measured at each compression-decompression cycle.

σ10%	FOAM	Si-SCD-M	C-SCD-M	C-APD-M	C-SCD	C-APD
Cycle 1 (kPa)	3.96	10.25	15.81	12.49	22.62	20.50
Cycle 2 (kPa)	3.88	10.20	15.67	12.11	21.84	20.03
Cycle 3 (kPa)	3.83	10.18	15.62	11.90	21.42	19.80
Cycle 4 (kPa)	3.79	10.15	15.54	11.75	21.16	19.62
Cycle 5 (kPa)	3.77	10.14	15.51	11.68	20.95	19.42
[(C1-C5)/C1] × 100	4.70	1.01	1.88	6.51	7.35	5.25

Table S3. Relative values of stress at 10 % of strain for all the samples measured at each compression-decompression cycle.

σ10% /ϱ	FOAM	Si-SCD-M	C-SCD-M	C-APD-M	C-SCD	C-APD
Cycle 1 (kPa·m^3/kg)	0.135	0.129	0.176	0.060	0.241	0.084
Cycle 2 (kPa·m^3/kg)	0.132	0.129	0.175	0.058	0.233	0.082
Cycle 3 (kPa·m^3/kg)	0.130	0.129	0.174	0.057	0.228	0.081
Cycle 4 (kPa·m^3/kg)	0.129	0.128	0.173	0.056	0.226	0.080
Cycle 5 (kPa·m^3/kg)	0.128	0.128	0.173	0.056	0.223	0.079
[(C1-C5)/C1] × 100	4.698	1.005	1.879	6.508	7.353	5.245
